# Sang Froid in a time of trouble: is a vaccine against HIV possible?

**DOI:** 10.1186/1758-2652-12-2

**Published:** 2009-02-02

**Authors:** Stanley A Plotkin

**Affiliations:** 1 University of Pennsylvania, Philadelphia, PA, USA; 2 Sanofi Pasteur, 4650 Wismer Road, Doylestown, PA 18902, USA

## Abstract

Since the announcement of the STEP trial results in the past months, we have heard many sober pronouncements on the possibility of an HIV vaccine. On the other hand, optimistic quotations have been liberally used, from Shakespeare's *Henry V*'s "Once more unto the breach, dear friends" to Winston Churchill's definition of success as "going from one failure to another with no loss of enthusiasm". I will forgo optimistic quotations for the phrase "Sang Froid", which translates literally from the French as "cold blood"; what it really means is to avoid panic when things look bad, to step back and coolly evaluate the situation. This is not to counsel easy optimism or to fly in face of the facts, but I believe that while the situation is serious, it is not desperate.

I should stipulate at the outset that I am neither an immunologist nor an expert in HIV, but someone who has spent his life in vaccine development. What I will try to do is to provide a point of view from that experience.

There is no doubt that the results of STEP were disappointing: not only did the vaccine fail to control viral load, but may have adversely affected susceptibility to infection. But HIV is not the only vaccine to experience difficulties; what lessons can we glean from prior vaccine development?

## Lessons from vaccinology

First, look at an uncomplicated example: the rubella vaccine. This is a live attenuated virus that was isolated in WI-38 fetal fibroblasts during the 1962/63 rubella pandemic and attenuated by low temperature passage in those same cells [1]. By selection of clones replicating at low temperature, we obtained a virus that consistently multiplied in seronegative humans and that evoked both humoral and mucosal immune responses that blocked superinfection [2]. Why was it successful in giving immunity? Of course, the answer is this: neutralizing antibodies to rubella present in the serum and on the mucosa are correlates of protection in preventing both nasopharyngeal implantation and subsequent viremia [3].

However, things are not always that easy. Take the paramyxoviruses measles and respiratory syncytial virus (RSV) as examples. Live measles virus has been a great success in eliminating the disease, but in the early days there was also a licensed killed measles vaccine. Unfortunately, when vaccinated children were exposed to wild measles they suffered an atypical disease that included severe pulmonary, hepatic and dermatologic manifestations. Similarly, a formalin inactivated RSV vaccine was tested in infants, many of whom developed severe respiratory disease after subsequent natural infection with the virus [4].

The pathogenetic features of these adverse reactions were similar [Table 1]. In both cases, the antibodies elicited had either disappeared or were non-protective because directed against the wrong protein, the T cell response was Th2 biased and contributed to the pathology, and replication of wild virus was enhanced [5-8]. Although I will not argue that this type of reaction could also explain the putative enhanced acquisition of HIV in the STEP and Phambili trials, it at least illustrates the idea that in the absence of functional antibodies, cellular immunity of the wrong type can enhance, rather than diminish susceptibility.

**Table 1:** Severe reactions to Inactivated Measles and RSV Vaccines

Following exposure, vaccinees had exaggerated disease in lungs.
Pathology included immune complex deposition and high replication in the lungs.

Vaccines elicited non-protective, low avidity, waning antibodies.

Vaccines elicited strong CD4+ proliferation with a Th2 cytokine response, including IL-13

Caused cessation of use of both vaccines

Another type of misadventure happened with the first licensed rotavirus vaccine. This was an orally administered mixture of a simian rotavirus and reassortants of human and simian rotaviruses in which the simian virus contributed 10 of the 11 double-stranded RNA segments. Although protective, it caused intussusception (intestinal invagination) in approximately one in 10,000 vaccinees [9]. This happened because the supposedly attenuated simian vector retained pathogenicity for the infant intestine, causing diarrhea and fever [10]. This problem was solved in my former laboratory by substituting a bovine rotavirus as vector, and in another lab, by classical attenuation of a human rotavirus [11,12]. Neither of the new vaccines causes intussusception [13,14]. The point is that the choice of a supposedly attenuated vector is a key issue, and that the wrong choice of vector brings safety problems.

Another lesson from vaccinology is that correlates of immunity may be complex, and antibody and cellular immunity are often collaborative. This point can be illustrated with reference to cytomegalovirus (CMV) [15,16]. As in HIV, superinfection may occur in previously infected individuals, but the course of secondary infection is much less pathogenic than in non-immune subjects. This is particularly important when infection occurs in pregnancy, as the fetal outcomes after primary or secondary infection are quite different.

Antibody against CMV alone may protect against primary infection, but if infection occurs, cellular immunity is critical in controlling it. In addition, challenge dose is an important variable, and can overcome moderate levels of immunity, a fact that may apply to HIV. This was shown by challenge studies in which seronegative volunteers could be infected with 10 PFU of a low-passage CMV, whereas naturally seropositive volunteers were protected against 100 PFU, but could also be infected if the dose was raised to 1000 PFU [17].

Nevertheless, two vaccines in development have shown moderate ability to prevent or modify CMV infection. One is based on a live attenuated virus, and one on a glycoprotein that induces neutralizing antibody [17,18]. Thus, the fact that superinfection has been demonstrated in some already HIV-infected people does not necessarily rule out a role for immunity in controlling disease after infection [19,20].

Another example is immunity to smallpox after vaccinia, about which one can say that antibody is key: high titers give sterile immunity. However, as antibody wanes, infection may occur, which CD8+ T cells must control. Antibody lasts forever after vaccination and CD4+ T cells last almost forever, but CD8+ T cells disappear after about 20 years. Thus, although complete protection is temporary, protection against severe disease is permanent [21].

The last agent I would like to discuss before turning to HIV is hepatitis C. There are many similarities between the two agents, including the rapid development of geographical variation, with a 30% nucleotide difference between hepatitis C genotypes [22]. Although hepatitis C is a flavivirus, it shares a number of properties with HIV, as shown in Table 2.

**Table 2:** Similarities between Hepatitis C and HIV

	HCV	HIV
Envelope and Core Ags	**+**	**+**
Glycosylated envelope protein	**+**	**+**
Envelope is neut. target, but hypervariable	**+**	**+**
Chronic viremia	**+**	**+**
Escape mutation	**+**	**+**
Geographical genetic variation	**+**	**+**
PD-1 Upregulated	**+**	**+**
High titer neutralizing Ab protects	**+**	**+**
Strong cell responses against multiple epitopes necessary for control of viremia	**+**	**+**
CD4+ cells needed to sustain CD8+ T cells	**+**	**+**
Tcm cells needed for long-term control	**+**	**+**

**Figure 1:**
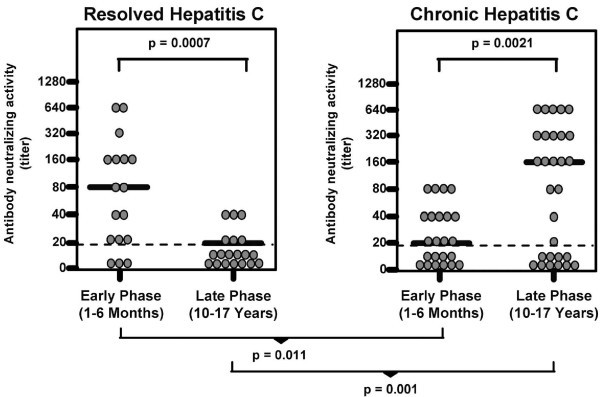
**Neutralizing antibodies in patients with resolved or chronic hepatitis C**.

Interestingly, patients who resolve acute hepatitis C infections have higher levels of neutralizing antibodies early in infection than do those who go on to chronic infection (Figure 1). As in the case of HIV, antibodies do not help when they develop late in chronic infection [23]. The target of neutralizing antibodies is the E2 envelope protein, and as in HIV, escape occurs [24,25].

However, it appears that late in the infection, induction or reconstitution of cellular immune responses also correlates with recovery from chronic hepatitis C viremia [26-30]. Those cellular responses are mediated by both CD4+ and CD8+ T cells directed against non-structural as well as structural proteins; to be effective, those responses must be strong, highly avid, and directed against multiple epitopes [31-34]. Although a crucial difference between the two viruses is the lack of integration by hepatitis C in contrast to HIV, I think it is instructive to see that a chronic infection can be counteracted by standard immune responses [32].

## Innate immunity

So what can be said about immune protection against HIV? With regard to innate immune responses, we know that they are clearly valuable [35,36], both immediately after infection and as adjuvants to adaptive immune responses. The question is: do they have memory? A method of maintaining elevated innate immune responses after immunization, particularly NK cells or intracellular APOBEC3G, could be valuable, although contrariwise after HIV infection, it has been reported that an HIV-induced ligand is responsible for CD4+ T cell destruction by NK cells [37].

On the other hand, a recent report [38] shows that the gene Apobec3 encodes Rfv3, which enhances neutralizing antibody responses against lentiviruses. This opens a new avenue of research to counteract its antagonist, the Vif element of HIV. In addition, soluble CD40 ligand has been shown recently to enhance HIV-specific memory T cell responses [39,40].

## Antibodies

Clearly, everybody would like to know how to induce a neutralizing response that covers primary isolates from all of the clades. A recent list of approaches is shown in Table 3[41]. To this list may be added: the recent studies attempting to mimic the b12-like antibodies produced by some infected individuals [42]; studies using alloantigens like hsp70 as part of an immunization regimen that apparently evokes a wider breadth of neutralization [43]; and the use of AAV as a vector to carry an antibody-producing gene into the cells of a vaccinee [44].

**Table 3:** Novel approaches to the design of envelope immunogens

Mimic native trimer on virion surface
Redirect immune responses to conserved conformational epitopes

Add disulfides or other amino acids to stabilize conformational epitopes

Bind envelope to CD4 or CD4-mimetic peptide

Remove carbohydrate residue or entire carbohydrate side chains

Redirect responses away from variable epitopes

Remove one or more variable loops
Add carbohydrate side chains to hide
variable regions

However, short of the ideal of broad neutralization with a single antigen, it is not beyond our abilities to produce multivalent vaccines. Because of multiple serotypes or subtypes, numerous licensed vaccines are actually multivalent, including those for Human Papilloma, Influenza, Meningococcal, Pneumococcal, Polio, and Rotavirus. Moreover, every year we change the valences of influenza vaccines to match the evolution of the virus. Although this is not an ideal scenario, most years it works well; on condition that surveillance is good, and that there are regional manufacturers, it is practical to make different vaccines for different areas.

Thus, although antibodies to conserve epitopes are highly desirable, antibodies to gp120 loops that mutate and are regional in distribution may require continuous updating and regionalization of vaccine antigens (as for flu), as well as the inclusion of multiple gp 120s. Even with breakthroughs in finding conserved epitopes, I doubt that we can escape totally from having to make multivalent or regional HIV vaccines [45]. Indeed, recent reports suggest that multivalent HIV envelopes do give broad neutralizing responses [46-49].

Does antibody protect against HIV infection? Clearly, non-human primate studies using HIVIG or monoclonal antibodies strongly support an affirmative answer [50-53]. In addition, the burden of evidence is in favor of a protective ability of maternal neutralizing antibodies in prevention of HIV transmission to the newborn [54]. Moreover, it has been reported that neutralizing antibodies develop rapidly and in high titer after HIV-2 infection, which could explain the much slower disease progression in HIV-2 patients [55-57].

How much antibody is needed for protection? A number of estimates have been made, and these are summarized in Table 4[50,52,58-61]. In addition, although superinfection is a fact in the presence of low levels of homologous neutralizing antibodies, there are data suggesting high levels are protective [62]. So if high levels of antibody are necessary for protection, in line with the need for multiple hits to neutralize the virion, and as HIV spreads from the site of implantation within several days, effector B cells must be in the circulation and producing antibody at the time of exposure [63,64]. Thus, booster doses of an AIDS vaccine will be necessary to maintain protective levels of antibody.

**Table 4:** Estimates of titers of neutralizing antibodies required for sterile protection against HIV.

Investigator	Titer	SN End Point	Species	Remarks
Trkola et al [58]	1/200	70%	Human	Acute infection
Parren et al [60]	1/400	90%	Macaques	SHIV Challenge
Nishimura et al. [61]	1/38	100%	Macaques	SHIV Challenge
Mascola et al [50,52]	1/50, 1/29–1/88	90%	Macaques	SHIV Challenge
Trkola et al [59]	1/400	90%	Human	Rebound after HAART

Indeed, booster doses are commonly needed for vaccines, even for some that are highly efficacious. They are almost always needed for inactivated vaccines, e.g. tetanus, diphtheria, and polysaccharide conjugates (exceptions are hepatitis A and hepatitis B), and are often needed for live vaccines, e.g. measles, mumps, and smallpox (exceptions are rubella and OPV). This may be an inconvenient truth, but the use of adjuvants might help prolong immunity.

The new adjuvants now available in vaccinology are legion, and they increase breadth as well as height of antibody responses. They include oil-in-water and water-in-oil emulsions, saponins, liposomes, lipopolysaccharides, cytokines, cationic polymers for DNA plasmids, mast cell activiators and numerous toll-like receptor (TLR) agonists. A recent report showed that an oil-in-water emulsion containing monophosphoryl lipid A and QS-21 substantially increased the number of primary isolates that could be neutralized in vitro by rabbit antisera [45,65]. Much more work is needed in this area [66-68].

## Cellular immunity

**Figure 2:**
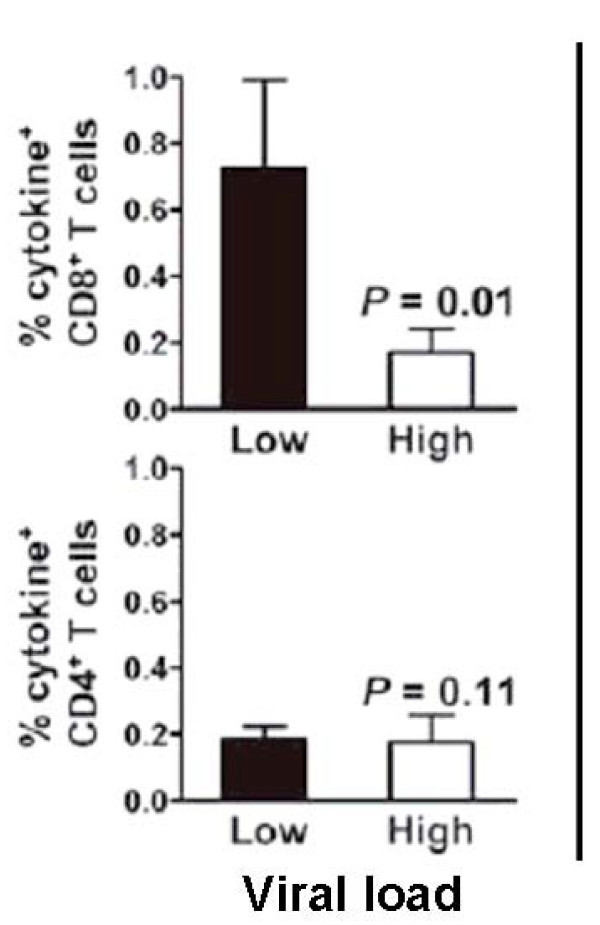
**Control of High SHIV Viral Load by CD8+ Cells After Vaccination**.

**Figure 3:**
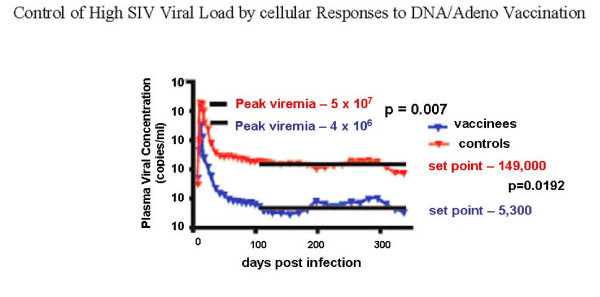
**Control of High SIV Viral Load by cellular Responses to DNA/Adeno Vaccination**.

I think it is safe to say that cytotoxicity mediated by CD8+ T cells can for a time suppress HIV viral load, even if it can not prevent acquisition of infection [69-79]. Clinical data correlating CTL responses with control of viral load and macaque studies by many labs have made that point clearly. Two examples are illustrative: in Figure 2, CD8+ T cells were clearly associated with low viral loads after challenge with SHIV [70]; and in a study of a DNA prime/adenovirus 5 boost, CTL against gag alone reduced viral load after SIV challenge (Figure 3). Moreover, among many other factors, elite HIV controllers, long-term non-progressors, and multiply exposed sex workers all have evidence of potent CD8+ T cells in the blood and in the mucosa [80-82], as well as innate immune factors [83].

However, there are issues of quantity. Recently, CTL responses were measured after two conventional live vaccines, smallpox and yellow fever [84]. In terms of percent CD8+ T cells specific to the vaccine, smallpox vaccine induced about 10% vaccinia-specific cells producing IFN-gamma, whereas yellow fever vaccine induced more than 2% yellow fever-specific cells. Compare those figures to the data from the STEP study in which only 0.5 to 1% of CD8+ T cells were specific to HIV (J McElrath, personal communication, 2008). Thus, it is legitimate to ask whether paucity of response played a role in the STEP failure.

Numerous factors influence the quality of CTL response, some of which are listed in Table 5. Many groups have demonstrated the importance of polyfunctionality, as defined by cytokine and chemokine secretion, in the control of HIV viral load [85-89] Other aspects of function that are suggested to be important include: CTL avidity [90,91]; number of epitopes seen by the CD8+ T cells [90,92], which is a reason for exploring the use of consensus and mosaic sequences to induce responses to more epitopes [86,91,93,94]; presence of polyfunctional CD8s in the rectal mucosa; preservation of Th17 cells [95]; and persistence of both CD4+ and CD8+ central memory T cells [92,96]. As it is likely that semen of HIV-transmitting patients will have both cell-free and cell-associated virus, it appears necessary that the CD8+ T cells be capable of killing infected cells in the inoculum [97].

**Table 5:** Cellular immune responses to HIV that could be improved

Quantity of specific CD8+ cells
Polyfunctionality of CD8+ cells

Avidity of CD8+ cells

Number of epitopes seen

Intestinal homing of CD8+ cells

Th17/Tregs balance

Increased CD4+ central memory cells

Increased CD8+ central memory cells

My goal here is not to exhaustively examine all of the important T cell responses, but rather to say that there are numerous leads with regard to improving cellular immune responses to an HIV vaccine, and that the failure of the first trial of this idea says only that the responses induced were inadequate to simulate those induced during natural infection that appear to control HIV temporarily.

## Mucosal immunity

It has become a cliché to say that vaccines can not provide sterile immunity. In my view, this is a canard. As indicated in Table 6, if the pathogenic agent is injected into the blood stream, as in arbovirus infection, or acts by a toxin, as in tetanus, sterile immunity is undoubted. In addition, if the agent implants first on the mucosa, as in many infections, sterile immunity is achievable on condition that mucosal immunity is sufficient to abort that replication. Examples of this principle include resistance to measles and rubella after vaccination with live viruses that induce both serum and mucosal antibody [21,98], and live or killed influenza vaccines, after which induction of either serum or mucosal antibody can completely prevent infection [99].

**Table 6:** Do vaccines elicit "sterile" immunity?

Yes	Depends Mucosal Presence of Antibody
Diphtheria	Polio
Hepatitis A	Hib
Hepatitis B	Influenza
Lyme	Measles
Rabies	Pertussis
Tetanus	Rubella
Yellow Fever	Varicella

Mucosal immunity is as complex as systemic immunity [100-104]. Secretory IgA may neutralize on the surface or block transcytosis [105]. Second, the CTL population in the intestine is numerous and can kill HIV-infected cells, which is important in view of the evidence that preservation of intestinal memory CD4+ T cells contributes to a good prognosis for the subsequent course of HIV infection [106,107]. Third, serum IgG can diffuse onto mucosal surfaces, particularly in the respiratory tract. The latter fact probably accounts for the reduction of pharyngeal carriage of encapsulated bacteria by conjugated polysaccharide vaccines, and the reduction of virus titer in the pharynx and stool of IPV vaccinees [21,102].

I alluded to the live, orally administered rotavirus vaccine previously, and there is another lesson to be learned from the rotavirus story: rotavirus diarrhea is caused by replication of the virus in intestinal cells. There are three important proteins of the virus: two of these, vp4 and vp7, induce neutralizing antibodies, whereas the third, vp6, induces non-neutralizing antibodies and cellular immune responses.

With regard to the correlates of immunity, efficacy studies show that type-specific neutralizing antibodies are an important factor in protection. However, studies of natural immunity show that non-neutralizing as well as neutralizing antibodies to vp6 also correlate with protection [108,109]. Moreover, studies in animals demonstrate that CTL in the intestinal lining against vp6 also have a role [110]. Finally, measurement of serum IgA antibodies provide a surrogate of protection by the vaccine [111], indicating that secretory IgA in the intestinal mucosa plays a major role in that protection [112].

**Figure 4:**
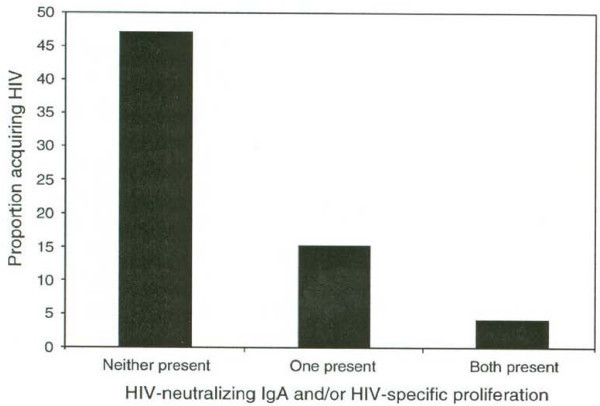
**Acquisition of HIV by Kenyan Sex Workers Prevented by Genital IgA and Systemic T Cell Proliferation**. Obviously, there are many problems to solve in attempting mucosal immunization. One approach is to mix routes of administration, for example priming with oral vaccination and following with parenteral boost. Moreover, it is not impossible to consider mixed intranasal and intrarectal administration to immunize both the genital and gastrointestinal tract. Aerosol administration of HPV vaccine has been reported to induce IgA secreting cells in the genital tract [115], and there is recent work suggesting that sublingual administration of antigens may be a way around compartmentalization of mucosal immunity [116] (see table 7).

Thus, mucosal immunity collaborates with other functions to control rotavirus, a theme reflected in studies of macaques and of Kenyan sex workers (Figure 4), in whom systemic T cell proliferation and neutralizing antibodies at the level of the genital and intestinal tracts were synergistic in protection against HIV [113,114].

Obviously, there are many problems to solve in attempting mucosal immunization. One approach is to mix routes of administration, for example priming with oral vaccination and following with parenteral boost. Moreover, it is not impossible to consider mixed intranasal and intrarectal administration to immunize both the genital and gastrointestinal tract. Aerosol administration of HPV vaccine has been reported to induce IgA secreting cells in the genital tract [115], and there is recent work suggesting that sublingual administration of antigens may be a way around compartmentalization of mucosal immunity [116] (see table 7).

**Table 7:** Some newer strategies for an HIV vaccine

Replicating vectors, e.g. Adenoviruses 4 and 7, CMV, Sendai, VSV, alphavirus-VSV
DNA plasmids with electroporation

Non-parenteral routes of administration: intranasal, rectal, sublingual

DNA/NYVAC prime boost regimen

Gene-driven HIV antibody

Anti-phospholipid antibodies

Live, attenuated HIV, e.g. Δnef, Δnef/vpr, ΔGY

(Canarypox/gp prime-boost 120 trial still ongoing in Thailand)

## The future

Of course, we must look at new vectors [117-122]. Replicating adenovirus vectors boosted by viral proteins have given promising results in prevention of SIV infection in macaques [123-125]. An interesting observation made in those studies and in other studies in macaques is the protection afforded by non-neutralizing antibodies through their action on infected cells by mechanisms such as ADCC [126-128]. This echoes the theme mentioned in relation to rotavirus.

Cytomegalovirus is under test as a replicating vector, as are measles, Sendai viruses and VSV [129,130]. DNA plasmids are enjoying a renaissance thanks to the concomitant use of electroporation and new adjuvants [49,131,132]. In addition, the European Consortium, has reported polyfunctional T cell responses in humans after a DNA-NYVAC vaccinia regimen [133]. Non-parenteral routes of administration of non-replicating vectors are also being explored [134]. Transfer of the gene for a neutralizing antibody via an adeno-associated virus vector to vaccinees is another intriguing approach [44]. Homology of anti-phospholipid antibodies and HIV epitopes is being explored [135]. And some of our hearts still belong to live attenuated HIV [136-138], although this is a contentious area owing to safety concerns. One should also keep in mind that the canarypox prime, gp120 boost trial in Thailand has survived analyses for the futility of efficacy and will be reported this year, and that the prime-boost concept using a DNA prime and Ad5 boost, which gives enhanced immune responses in comparison with Ad5 alone, remains to be tested in the clinic [139].

In summary, I believe that an effective HIV vaccine will need to stimulate neutralizing antibodies, as well as CD4+ and CD8+ cellular responses in the blood and on the mucosa. This is hardly a novel conclusion, and it is a tall order, but the biology of the virus and the history of vaccinology tell us, respectively, that those responses are necessary and that they have been feasible to induce for previous vaccines.

At the beginning of this article, I disdained the use of optimistic or pessimistic quotations to justify opinions about the future of HIV vaccine development. I have tried to be realistic in my own assessment of the situation and I will close with one quotation, because it is definitely realistic, as everyone who has ever worked in a laboratory knows. It comes from Emile Roux, the associate of Pasteur and a brilliant scientist in his own right. He said, "Science appears calm and triumphant when it is completed; but science in the process of being done is only contradiction and torment, hope and disappointment." Let us not give up, for as Roux would agree, the goal is worth it.

## Competing interests

The author is a paid consultant to Sanofi Pasteur, Merck, and other vaccine manufacturers.
